# ^99m^Tc-NTP 15-5 is a companion radiotracer for assessing joint functional response to sprifermin (rhFGF-18) in a murine osteoarthritis model

**DOI:** 10.1038/s41598-022-11080-4

**Published:** 2022-05-17

**Authors:** Arnaud Briat, Claire Jacques, Mélodie Malige, Laure Sudre, Geoffroy Nourissat, Philippe Auzeloux, Hans Guehring, Florent Cachin, Francis Berenbaum, Elisabeth Miot-Noirault

**Affiliations:** 1 Clermont Auvergne University, INSERM U 1240 Molecular Imaging and Theranostic Strategies, F-63000 Clermont-Ferrand, France; 2 Sorbonne University, INSERM U 938 CRSA, Paris, France; 3 Sorbonne University, INSERM U 938 CRSA, Clinique des Maussins, Groupe Ramsay Générale de Santé, Paris, France; 4 Global Clinical Development, Merck KGaA, Darmstadt, Germany; 5 Jean Perrin Cancer Centre, Nuclear Medicine Department, F-63000 Clermont-Ferrand, France; 6 Sorbonne University, INSERM U 938 CRSA, AP-HP Saint-Antoine Hospital, Paris, France

**Keywords:** Imaging, Molecular imaging, Drug discovery, Diagnostics

## Abstract

With the emergence of disease modifying osteoarthritis drugs (DMOAD), imaging methods to quantitatively demonstrate their efficacy and to monitor osteoarthritis progression at the functional level are urgently needed. Our group showed that articular cartilage can be quantitatively assessed in nuclear medicine imaging by our radiotracer ^99m^Tc-NTP 15-5 targeting cartilage proteoglycans. In this work, surgically induced DMM mice were treated with sprifermin or saline. We investigated cartilage remodelling in the mice knees by ^99m^Tc-NTP 15-5 SPECT-CT imaging over 24 weeks after surgery, as wells as proteoglycan biochemical assays. OA alterations were scored by histology according to OARSI guidelines. A specific accumulation of ^99m^Tc-NTP 15-5 in cartilage joints was evidenced in vivo by SPECT-CT imaging as early as 30 min post-iv injection. In DMM, ^99m^Tc-NTP 15-5 accumulation in cartilage within the operated joints, relative to contralateral ones, was observed to initially increase then decrease as pathology progressed. Under sprifermin, ^99m^Tc-NTP 15-5 uptake in pathological knees was significantly increased compared to controls, at 7-, 12- and 24-weeks, and consistent with proteoglycan increase measured 5 weeks post-surgery, as a sign of cartilage matrix remodelling. Our work highlights the potential of ^99m^Tc-NTP 15-5 as an imaging-based companion to monitor cartilage remodelling in OA and DMOAD response.

## Introduction

Osteoarthritis (OA) is a slowly, progressive, ultimately degenerative disorder of movable joints, mainly identified at the clinical level by pain and functional limitation and involving all joint structures^1^. OA progression can be characterized by joint cartilage degradation and loss, subchondral bone remodelling, and synovial membrane inflammation. Current treatments can improve symptoms but do not delay the progression of disease, the total joint replacement finally becoming the only possible outcome for many patients^1^.

The articular cartilage is responsible for the biomechanical properties of the joint, supported by an extracellular matrix (ECM), rich in fibrillar proteins such as collagens, and in proteoglycans (PG). PG contain covalently bound glycosaminoglycans (GAGs), which are essential to the function of the molecule as they draw water into the cartilage matrix, giving it the ability to withstand compression^2^. During OA development, a loss of PG and a disruption of the collagen network occur, leading to the matrix destruction and ultimately to the total cartilage loss^3^. Therefore, the articular cartilage and its ECM appear as therapeutic targets of importance.

With the better understanding of the pathophysiology of OA progression, promising therapeutic targets have been identified, with the emergence of Disease Modifying OsteoArthritis Drugs (DMOAD), aiming at not only assisting with symptom management but also modifying the structural course of the disease^4^. Despite extensive research on DMOAD, there is currently no pharmacological intervention approved for use in Europe or the USA demonstrating efficacy for modifying OA progression^5,6^. Even if promising DMOADs have emerged, the major challenge in demonstrating the proof of concept is to overcome (i) the absence of a precise assessment of the disease, particularly in the early stages, and (ii) the lack of consensus on how to detect joint changes and link them to clinically meaningful endpoints. Therefore, new treatments are needed, as well as new methods to evaluate their efficacy and to monitor OA progression. Authors indeed consider that research and development towards DMOADs is hampered by the lack of specific and sensitive imaging tools to reliably quantify OA progression, and monitor response to therapy, including radiographs and MRI^7,8^. Higher sensitive imaging approaches appear of real need to evaluate OA progression and to monitor response to innovative therapies^8^.

Few DMOAD currently under development are aiming at preventing cartilage deterioration and/or restoring cartilage thickness^5,6^. Among them, sprifermin, a recombinant human Fibroblast Growth Factor 18 (rhFGF-18), appeared as the most promising molecule. In rats^9^ and in human explants^10,11^, it has been demonstrated that FGF-18 increased chondrocytes proliferation, as well as ECM deposition and PG production, leading to an increase in cartilage thickness. The strong anabolic effect of sprifermin seems to follow a sequential process, with an early increase of aggrecanase activity leading to aggrecan degradation, described as a prerequisite for cell proliferation, followed by ECM molecules production by newly produced chondrocytes, including sustained PG production, and ultimately cartilage regeneration^11^. To date, sprifermin is the only candidate DMOAD with a proven structural effect on cartilage. The clinical relevance of the results obtained is currently being discussed^12–14^. This makes it the only possible gold standard to date for a study to investigate a new surrogate marker of cartilage remodelling.

In such a context, our group aims at validating a nuclear medicine imaging strategy targeting cartilage PG in vivo, which could provide a functional access to joint and a new method to evaluate innovative therapies. Because cartilage contains up to 10% PG consisting of mainly chondroitin sulphate aggrecan which chains are negatively charged^15^, our strategy is based on the use of a bi-functional agent, the radiotracer ^99m^Tc-N-(triethylammonium)-3-propyl-[15]ane-N5 (^99m^Tc-NTP 15-5), that contains in its structure a positively charged quaternary ammonium function for binding to PG and a polyazamacrocycle to complex ^99m^Tc. Based on many preclinical studies, we believe that ^99m^Tc-NTP 15-5 and in vivo functional imaging of PG could provide a suitable set of criteria for quantifying cartilage functionality, and the efficacy of new emerging therapeutic strategies^16–21^.

In this study, we determined the relevance and sensitivity of ^99m^Tc-NTP 15-5 imaging in the destabilization of the medial meniscus osteoarthritis murine model, for assessing the structural effect of sprifermin, with the opportunity to bridge the gap between preclinical and clinical testing.

## Results

### SPECT functional cartilage imaging with ^99m^Tc-NTP 15-5 in healthy mice (Fig. 1)

**Figure 1:**
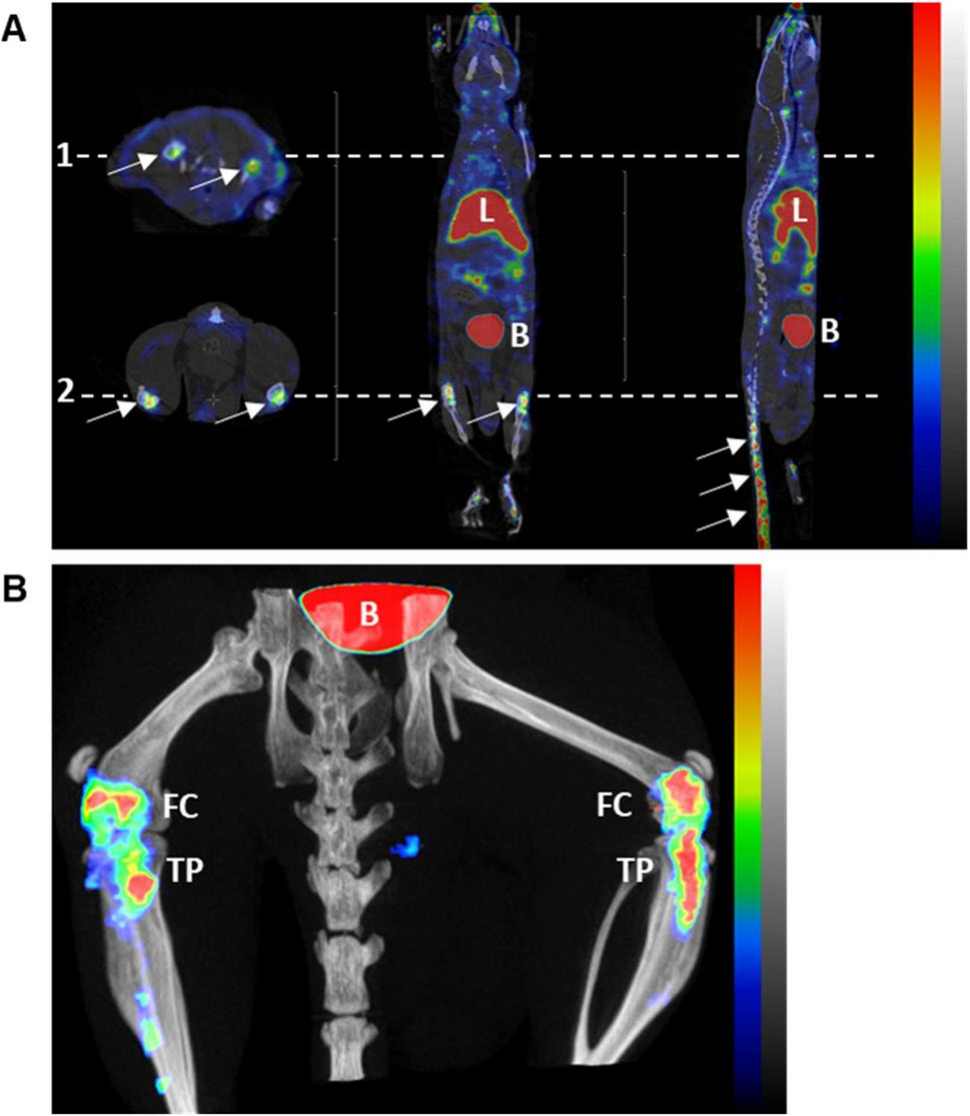
In vivo distribution by SPECT-CT imaging of ^99m^Tc-NTP 15-5 30 min after intravenous injection of 20 MBq of radiotracer in healthy mice. (**A**) Representative transversal, coronal, and sagittal slices of ^99m^Tc-NTP 15-5 distribution in anesthetized mice. From left to right: transversal views at the humeral head level (dotted line 1) and at the knees level (dotted line 2); coronal and sagittal whole-body views. Arrows indicate cartilage uptake in the mouse shoulders, knees, and intervertebral disks, respectively. Non-specific uptake is observed in the liver (L) and bladder (B). SPECT scaling range is 65 to 650 kBq/ml. (**B**) Representative SPECT-CT Maximum Intensity Projection 3D image focused on knees. Specific uptake in femoral condyle (FC) and tibial plateau (TP) is observed, as well as non-specific accumulation in bladder (B). SPECT scaling range is 650 to 2000 kBq/ml.

^99m^Tc-NTP 15-5 accumulation was evidenced from 30 min post-injection in many articular structures of the animals, such as knees, humeral head, and intervertebral disks (Fig. 1A). As evidenced on the coronal and sagittal slices, nonspecific accumulation was observed in liver, digestive tract, and bladder. At the knee level, scintigraphic images allow the discrimination of femoral condyle uptake from that of tibial plateau (Fig. 1B).

### Characterization of OA model: histology (Fig. 2)

**Figure 2:**
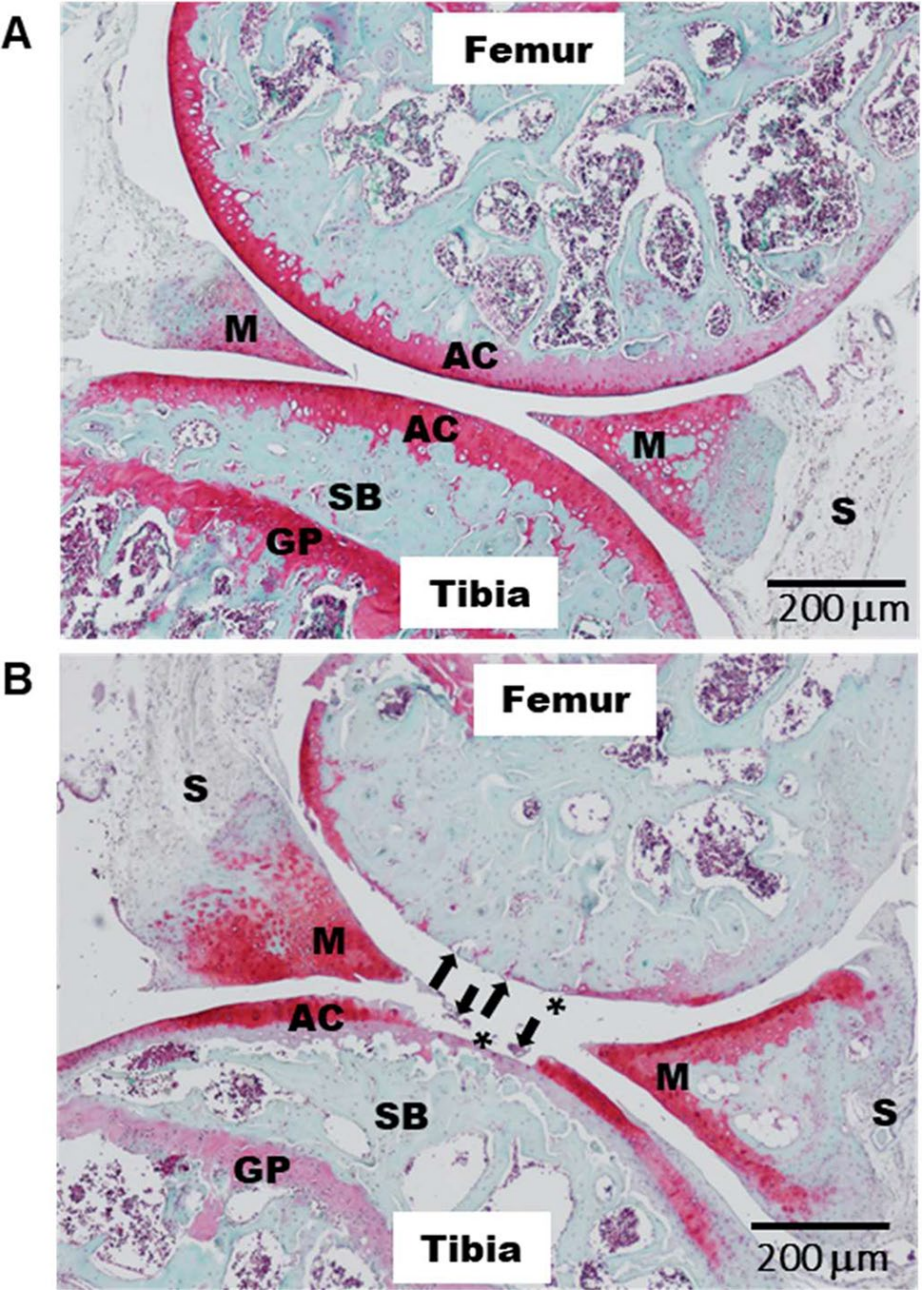
Characterization of in vivo mouse OA model with typical histological appearance: Representative haematoxylin-safranin-O-stained sections of knee from control (**A**) and DMM (**B**) mice respectively at 12 W after surgery. In (**B**), clear histopathological alterations: arrows indicate cartilage fibrillation, and asterisks indicate loss of cartilage. Scale bar: 200 µm; original magnifications 10×; AC, articular cartilage; M, meniscus; S, synovium; SB, subchondral bone; GP, growth plate.

OA defects in DMM mice were confirmed by histology 12 weeks following DMM surgery. As illustrated in Fig. 2, cartilage erosion was clearly evidenced on the tibial plateau as a decrease in thickness and proteoglycan loss as a staining intensity decrease (Fig. 2).

### Monitoring of ^99m^Tc-NTP 15-5 functional imaging of DMM mice submitted to sprifermin treatments (Fig. 3)

**Figure 3:**
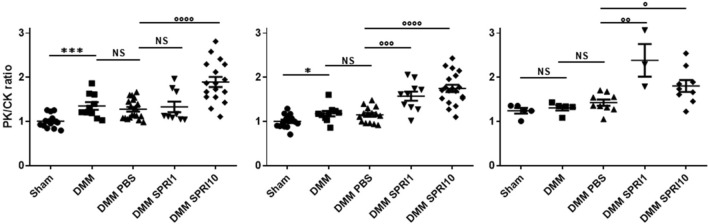
Time course of ^99m^Tc-NTP 15-5 ratios (PK/CK ratio = uptake of pathological knee/uptake of contralateral knee) in Shams, DMM, DMM + PBS, DMM + 3 × 1 µg sprifermin (DMM SPRI1) and DMM + 3 × 10 µg sprifermin (DMM SPRI10) at W7 (left), W12 (middle) and W24 (right) post-surgery. Results are mean ± SEM. DMM *versus* Sham: *p < 0.05, ***p < 0.001; DMM sprifermin *versus* DMM PBS: °p < 0.05, °°p < 0.01, °°°p < 0.001, °°°°p < 0.0001; NS: non-significant.

A differential accumulation of ^99m^Tc-NTP 15-5 occurred in the pathological knees (PK) compared to the contralateral knee (CK), at 7 W post-surgery with a significant (p = 0.0006) increase in radiotracer PK/CK ratio in DMM (1.35 ± 0.07) *versus* Shams (1.00 ± 0.04) (Fig. 3). This difference is still significant at 12 W, but not at 24 W. When DMM mice were treated by sprifermin (3 × 10 µg), PK/CK ratios were significantly higher than controls from 7 W (DMM SPRI10 *versus* DMM PBS, 1.89 ± 0.12 and 1.27 ± 0.04 respectively, p < 0.0001) to W24 (DMM SPRI10 *versus* DMM PBS, 1.80 ± 0.13 and 1.42 ± 0.04 respectively, p = 0.0234). When sprifermin was given at 1 µg per injection, increase in PK/CK ratio was statistically significant respectively to controls at later stages (12 W) (DMM SPRI1 *versus* DMM PBS, 1.57 ± 0.10 and 1.15 ± 0.04, p < 0.0003 at 12 W and 2.38 ± 0.37 and 1.42 ± 0.05 respectively, p = 0.0018 at 24 W). Intraarticular injections of PBS did not induce changes in ^99m^Tc-NTP 15-5 accumulation compared to DMM mice that were not injected (p = 0.4349, p = 0.7175, p = 0.2787 at 7 W, 12 W and 24 W respectively).

### Histological analysis of sprifermin effect in mice knees (Figs. 4 and 5)

**Figure 4:**
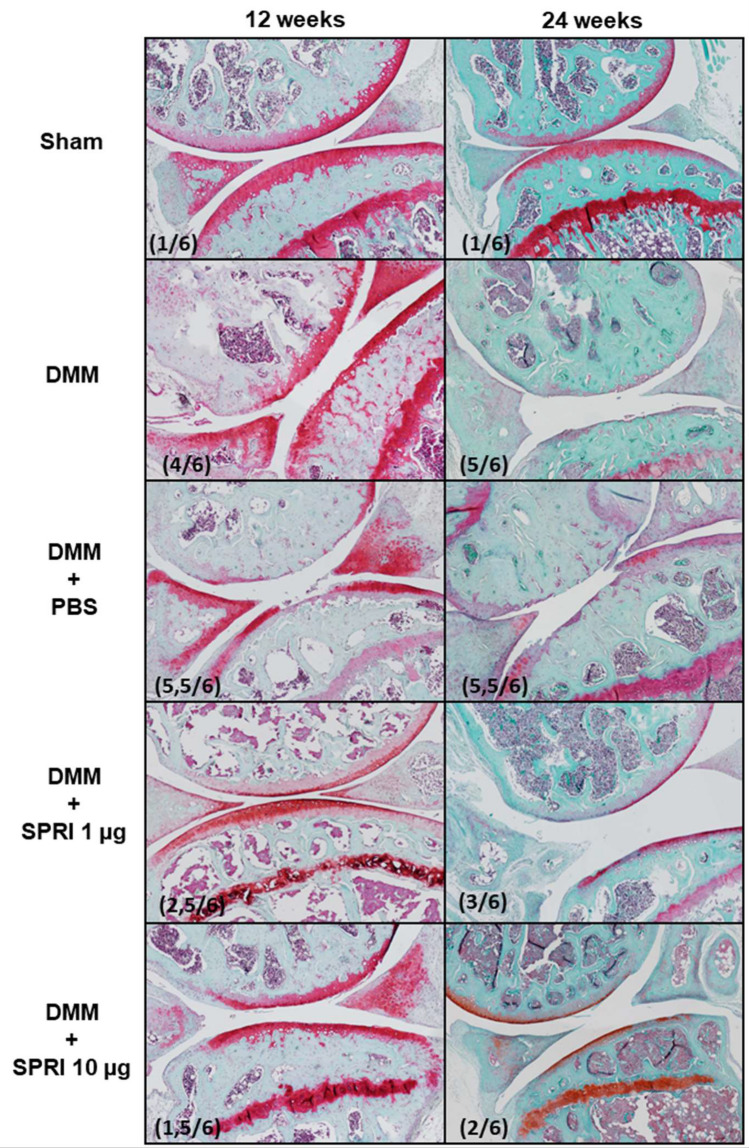
A representative histological sections from Shams, DMM, DMM + PBS, DMM SPRI1 and DMM SPRI10 at 12 W (left) and 24 W (right) after surgery. OARSI scores are indicated into brackets.

**Figure 5:**
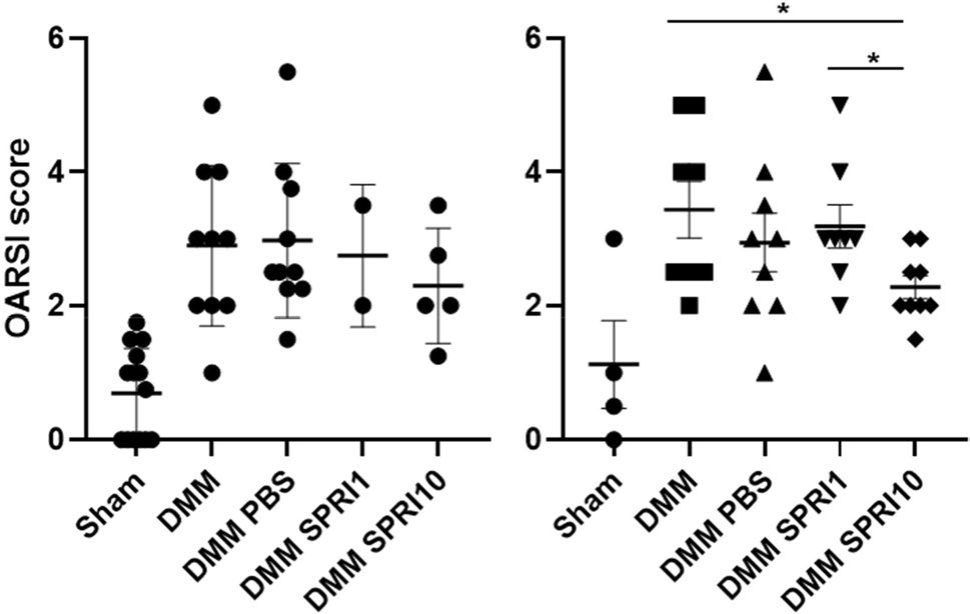
OARSI scores of Shams, DMM, DMM + PBS, DMM SPRI1 and DMM SPRI10 at 12 W (left) and 24 W (right) after surgery. Results are mean ± SEM. *p < 0.05 for DMM SPRI10 *versus* DMM SPRI1 and DMM.

OARSI scores have been determined from histological sections of the whole knees of each group. No noticeable alterations were observed in shams. DMM mice exhibited joint alterations with fibrillations and cartilage erosion, with a significant increase in OARSI scores compared to Shams at 12 W (2.90 ± 0.37 v*ersus* 0.69 ± 0.18, p < 0.0001) and at 24 W (3.43 ± 0.43 *versus* 1.12 ± 0.66, p = 0.012).

For DMM SPRI10, mean OARSI scores were lower respectively to controls at 12 W: scores of 2.3 ± 0.38 for DMM SPRI10 versus 2.90 ± 0.37 for DMM (p = 0.3389) and 2.97 ± 0.36 for DMM PBS (p = 0.269). This was more pronounced at 24 W with values of 2.28 ± 0.17 for DMM SPRI10 respectively to 3.43 ± 0.43 for DMM (p = 0.018) and 2.94 ± 0.44 for DMM PBS (p = 0.17).

For DMM SPRI1, mean OARSI scores (2.75 ± 0.75 at 12 W, and 3.18 ± 0.32 at 24 W) were not statistically different from scores in DMM (12 W: 2.90 ± 0.38, p = 0.873; 24 W: 3.43 ± 0.43, p = 0.649) and DMM PBS (12 W: 2.97 ± 0.36, p = 0.804; 24 W: 2.94 ± 0.41, p = 0.668).

Interestingly, at 24 W, OARSI score was significantly different (p = 0.0218) between the two doses of treatment of sprifermin: 2.28 ± 0.17, for DMM SPRI10 *versus* 2.94 ± 0.44, for DMM SPRI1.

### Proteoglycan content and ^99m^Tc-NTP 15-5 uptake in DMM PBS and DMM SPRI10 (Fig. 6)

**Figure 6:**
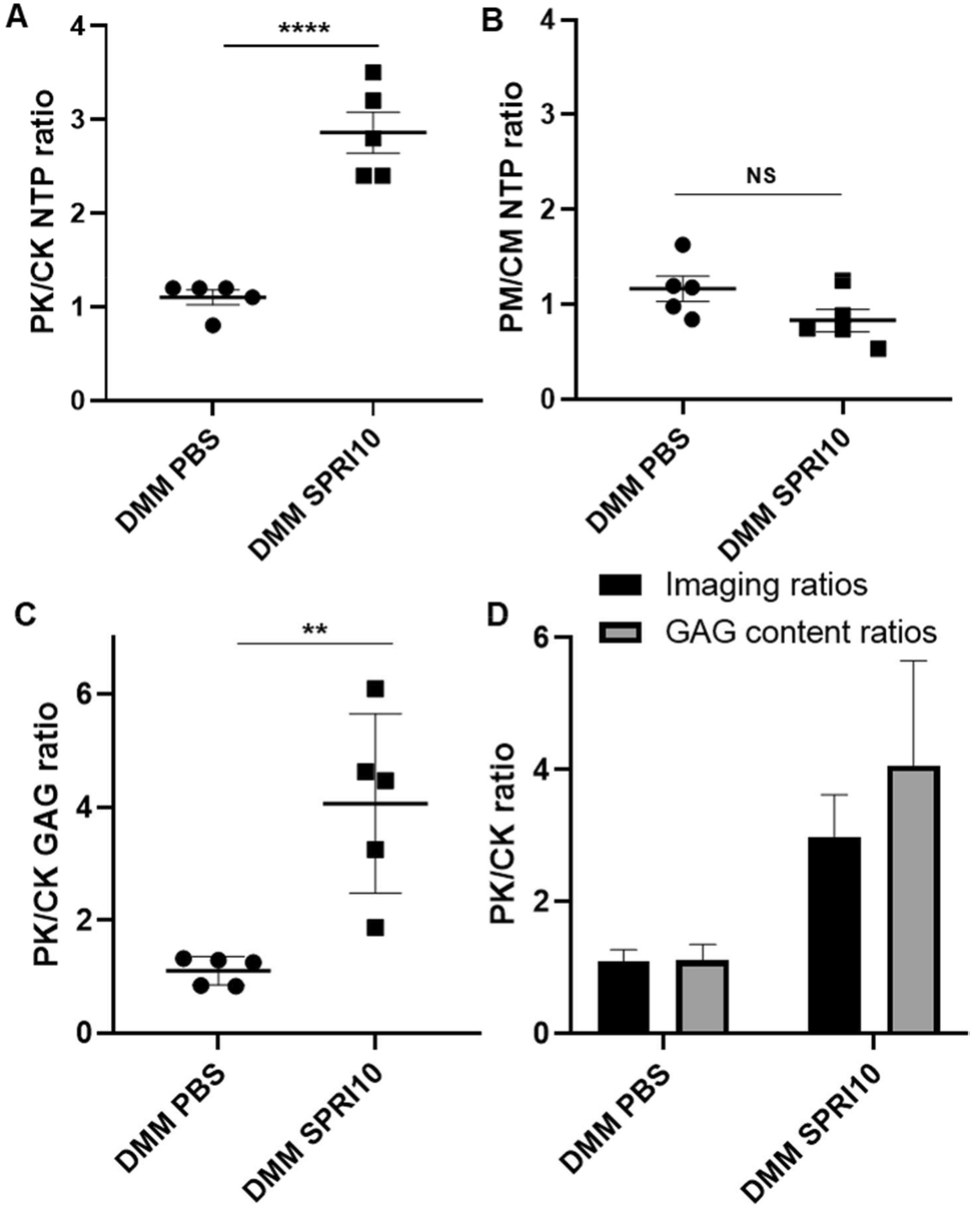
Sprifermin effect on imaging ratios and GAG content in DMM PBS and DMM SPRI10 at 5 W after surgery. (**A**) and (**B**): ^99m^Tc-NTP 15-5 ratios in knees (PK/CK ratio = uptake of pathological knee/uptake of contralateral knee) and muscles (PM/CM ratio = uptake in pathological leg muscle/uptake of contralateral leg muscle). (**C**) whole knee glycosaminoglycans content PK/CK ratios. (**D**) Correlation data summary. Results are mean ± SEM. DMM SPRI10 versus DMM PBS, ****p < 0.0001, **p < 0.01, NS: non-significant.

An additional protocol was especially dedicated to compare, in the same animals, imaging parameters and proteoglycan dosage of cartilage at 5 weeks after surgery. PK/CK radiotracer ratio was significantly higher in the sprifermin group compared to the PBS group (2.86 ± 0.22 *versus* 1.1 ± 0.08, p < 0.0001, Fig. 6A), whereas no change was evidenced in muscle uptake (Fig. 6B). Operated joints of DMM SPRI10 showed higher PG content than DMM PBS, with PK/CK ratios of 4.06 ± 0.71 and 1.11 ± 0.11 respectively (p = 0.0023; Fig. 6C).

## Discussion

^99m^Tc-NTP 15-5 was previously demonstrated to be a candidate for the in vivo functional nuclear imaging of proteoglycan remodeling in degenerative pathologies of cartilage^16–21^. In the present study, we evaluated in the surgically induced DMM model the relevance of ^99m^Tc-NTP 15-5 nuclear imaging for monitoring in vivo cartilage remodelling in response to sprifermin (rhFGF-18), an anabolic agent which previously demonstrated cartilage repair properties^9–14^. The surgical model of destabilization of the medial meniscus (DMM) has become a gold standard for studying the onset and progression of osteoarthritis^22^. Some studies reported mild cartilage lesions in mice as early as 2 weeks after surgery, with regional proteoglycan loss, chondrocyte clustering and osteophyte formations, with lesions being deeper into the calcified zone 10 weeks after surgery^23^. In our work, joint alterations were scored at 12- and 24-weeks following surgery according to OARSI recommendations^24^ and confirmed OA features in DMM, with OARSI scores higher than controls (Fig. 2, 4 and 5). In the DMM model, ^99m^Tc-NTP 15-5 accumulation in cartilage within the operated knee, relative to the contralateral one, was observed to change in the same animals as pathology progressed: PK/CK ratios of radiotracer uptake increased in the DMM group compared to the sham group at 7- and 12-weeks. Since ^99m^Tc-NTP 15-5 has been previously demonstrated, by Surface Plasmon Resonance to specifically bind to aggrecan (unpublished data) and was evidenced by autoradiographic studies to accumulate in knee cartilage of guinea pigs^16^, we consider that ^99m^Tc-NTP 15-5 imaging provides the assessment of joint integrity at the PG level. Such PK/CK scintigraphic ratio increase after surgery agrees with previously published results from our group in the meniscectomy and papain models of OA^16,25^. Hypertrophic changes in PG have been widely described in OA cartilage as a first line reparative reaction via an early increased synthesis of extracellular matrix (ECM) in damaged areas being followed at a later stage by degenerative destruction^26–31^.

We then evaluated sprifermin treatment on OA evolution in DMM treated at doses of 1- or 10-µg respectively to DMM receiving PBS, DMM without any treatment, and shams. Although not significant at 12 weeks, mean OARSI score in the DMM SPRI10 group was lower than DMM, DMM PBS and DMM SPRI1 (only 2 mice were analyzed in this group). At 24 W post-surgery, mean OARSI score was statistically lower for DMM SPRI10 than for DMM SPRI1 and DMM.

Histology of joints evidenced in both DMM and DMM PBS groups, at 12 W and 24 W, noticeable cartilage cauterization, and massive proteoglycan degradation. Compared with the other groups, the sprifermin groups (1 and 10 µg) demonstrated smoother surface of cartilage that appeared to be more pronounced for the highest dose. Such effects are consistent with previously published results in rats which demonstrated that intra-articular injection of sprifermin induced dose-dependent increase in overgrowth of new cartilage and in extracellular matrix production^9^.

The progressive changes in PG content of the extracellular matrix were monitored in vivo by ^99m^Tc-NTP 15-5 SPECT-CT imaging at 7-, 12- and 24-weeks post-surgery. An increase in PK/CK ratio was observed in sprifermin treated DMM respectively to the other DMM groups. From W7, sprifermin (3 × 10 µg) induced a significant increase of ^99m^Tc-NTP 15-5 ratios respectively to DMM PBS, as a sign of early cartilage remodelling. Considering DMM SPRI1, scintigraphic ratio increase became statistically different from controls only from 12 W. This tends to suggest that sprifermin effect on tracer accumulation is related to the dose that was administered. From these results, it seems interesting to refer to those of Moore and al. obtained in a rat model of OA and observing a significant effect of sprifermin on cartilage thickness at W6 after intra-articular injections of sprifermin, 5 µg bi-weekly over 3 weeks, while no significant effect was observed with the 1-µg dose^9^. DMM SPRI1 showed a higher mean effect on scintigraphic ratio at 24 W. However, differences were not statistically significant. It would be of interest to increase the DMM SPRI1 sample size to further study this.

Finally, using a dedicated cohort of mice receiving sprifermin 10 µg or PBS, imaging parameters and joints PG content were determined on the same animals, at 5 weeks after DMM surgery. For these animals, increase in scintigraphic ratios in response to sprifermin was concomitant with proteoglycan ratio increase. Such relation between ^99m^Tc-NTP 15-5 scintigraphic ratio and proteoglycan content was previously demonstrated in an experimental model of rheumatoid arthritis, under meloxicam treatment thus reinforcing the specificity of ^99m^Tc-NTP 15-5 to monitor the effect of treatments as well as monitoring disease progression^21^.

From our results, ^99m^Tc-NTP 15-5 radiotracer demonstrated its ability to assess in vivo cartilage repair by the anabolic agent Sprifermin, which has emerged as one of the most promising DMOAD^12–14,32–34^. For clinical development programs for DMOADs, the current regulatory guidance determined that approval requires inhibition of loss in joint space width (JSW) on plain radiograph, associated with symptomatic benefit^35,36^. ^99m^Tc-NTP 15-5 imaging has the opportunity to be a complementary method to conventional anatomical imaging such as radiography and MRI, used in routine to evaluate loss in JSW and cartilage. However, due to the slow progression of the disease, these anatomical techniques have a limited value for short duration DMOAD trials^37^.

For many years, nuclear medicine applications for OA progression evaluation have been under investigations^38^. To our knowledge, the radiotracers currently available for clinical OA imaging provide indirect evaluations of the pathology, such as bone and inflammation, which are useful for guiding choice of therapy but not for providing an early index of OA. Using PET in patients, ^18^F-NaF has recently been used to assess bone remodelling in knees of healthy and OA subjects^37^ and has been suggested for the evaluation of bone involvement in OA progression, in complement of other imaging techniques, such as MRI, which could measure other parameters of interest (cartilage thickness). ^18^F-Fluoro-Deoxy-D-Glucose (^18^F-FDG) has also been proposed to investigate metabolic changes in knee OA^39^ and to identify inflammatory regions of the joints in symptomatic patients^40^, thus limiting its potential applications to symptomatic OA. Regarding SPECT-CT studies, many addresses quantitative bone metabolism imaging with ^99m^Tc-DPD uptake being highly correlated with other imaging parameters for OA activity of the medial compartment of the knee^41^. Other radiotracers have been proposed to monitor the progression of OA disease. Activated macrophages were observed in rat models of OA using a folate receptor targeting radiotracer and SPECT^42,43^. Sobal et al. demonstrated increased joint to background ratio of ^99m^Tc labelled chondroitin sulphate (^99m^Tc-CS, which accumulates in cartilage inversely to negative charges of GAGs present in the ECM of the tissue) in dogs with OA with a significant positive correlation between grade of disease and scintigraphic ratio^44^. In this context, SPECT-CT imaging with ^99m^Tc-NTP 15-5 offers the unique advantage to be directly related to the negatively charged GAGs of PG^16–21^, responsible for the fixed charged density involved in biomechanical properties of articular tissue^45^.

### Conclusions

Our experimental results in the DMM model of OA treated by sprifermin demonstrate the proof of concept that ^99m^Tc-NTP 15-5 imaging could provide an early and sensitive tool to evaluate cartilage remodelling and DMOAD response.

These promising results are in favour of reinforcing first into humans transfer for evaluating the sensitivity of ^99m^Tc-NTP 15-5 imaging in patients with OA, that we have recently initiated (NCT04481230).

## Methods

### Animals

Animal experiments were conducted in compliance with the 2010/63/UE European Directive and with the relevant guidelines and regulations. In addition, all experiments were authorized by the French Ministry of Research after approval by the local ethical committee of Clermont-Ferrand (C2EA-02, approval n°04.623.02 and n°12160-2017111317154154v3). Experiments were conducted on a total of 104 C57BL/6j male mice (Charles River Laboratories). Mice (12 weeks old at time of surgery) were housed in standard conditions (n = 5 per cage, with enrichment), in ventilated racks, with a 21–24 °C temperature comprising 60% humidity and a 12 h light / 12 h dark cycle with access to food and water ad libitum. Animal health and wellbeing was monitored daily by competent persons. Each cage was allocated to a single treatment group. In addition, mice were ear marked under general gas anaesthesia using isoflurane at 2.5 *per* cent and 2:3 oxygen. All intravenous injections were made in lateral tail vein of vigil mice. For in vivo imaging, mice were placed under general gas anaesthesia. Euthanasia was performed by cervical dislocation after isoflurane gas overdose.

### Destabilization of medial meniscus experimental OA model

The DMM surgery has been performed according to already described procedures^46^. Briefly, a skin incision was performed, before opening the right knee joint capsule of the hind legs. The medial meniscus of the right knee was destabilized by cutting the medial menisci-tibial ligament. The "sham" intervention corresponds to the same procedure without meniscal destabilization. Afterwards, skin was closed with stitches, the joint capsule healing spontaneously. During the procedure, mice were placed under general gas anaesthesia (induction with isoflurane 4%, then 1.5% during the procedure). Anaesthesia induction was followed by an intraperitoneal injection of Buprenorphine (Buprecare, 0.05 mg/kg) for analgesia induction. Buprenorphine injections were performed following surgery every 8–12 h for 24–48 h.

### Study design

#### Model characterization

A first cohort of mice was used to verify the phenotype of DMM mice on knee joint lesions accordingly to the paper of Sonia Glasson^24^. We characterized osteoarthritic structural lesions in the DMM model at 12 weeks post-surgery using histology:

- Shams: n = 1 experiment corresponding to a total of 6 animals,
- DMM: n = 1 experiment corresponding to a total of 8 animals.

#### Sprifermin therapeutic protocol

Sprifermin was administered 2 weeks after OA surgical induction by injections into the joint cavity of knees through a 33-G needle of either 1 or 10 µg in 4 µl Phosphate Buffer Saline (PBS) over 3 weeks (1 injection per week, i.e., up to 5 weeks post DMM surgery). As a control for treatment, PBS alone was administered according to the same schedule as sprifermin.

#### Monitoring of sprifermin effect

The monitoring of sprifermin effect using SPECT-CT imaging and histology started 7 weeks post-surgery, i.e., 2 weeks after the end of treatment, and ended 24 weeks post-surgery. Two independent experiments were conducted, except for the sprifermin 1 µg condition. At initiation of each experiment, animals were randomly divided in 5 groups to have large enough data set to determine statistical significance up to 24 weeks. Groups were set as follows:

- Shams: n = 2 experiments corresponding to a total of 15 animals,
- DMM without any treatment: DMM, n = 2 experiments corresponding to a total of 15 animals,
- DMM with PBS treatment: DMM PBS, n = 2 experiments corresponding to a total of 20 animals,
- DMM with sprifermin treatment at 1 µg per injection: DMM SPRI1, n = 1 experiment corresponding to a total of 10 animals,
- DMM with sprifermin treatment at 10 µg per injection: DMM SPRI10, n = 2 experiments corresponding to a total of 20 animals.

Mice were imaged at 7-, 12- and 24-weeks post DMM surgery. Among these mice, a total of 12 Shams, 10 DMM, 19 DMM PBS, 10 DMM sprifermin 1 µg, and 14 DMM sprifermin 10 µg were used for histology and OARSI scoring at 12- and 24-weeks post-surgery.

#### Proteoglycan content and imaging

An additional protocol was especially dedicated to compare in the same animals, imaging parameters and proteoglycan dosage 5 weeks after surgery. A total of 10 animals was included in this cohort, consisting of:

- DMM with PBS treatment: n = 1 experiment corresponding to a total of 5 animals,
- DMM with sprifermin treatment at 10 µg per injection: n = 1 experiment corresponding to a total of 5 animals.

### In vivo SPECT-CT functional imaging of joint with ^99m^Tc-NTP 15-5

SPECT-CT imaging was performed 30 min after the intravenous injection of 20 MBq of ^99m^Tc-NTP 15-5. Multimodal SPECT-CT imaging was performed using a NanoScan SPECT-CT camera (Mediso Ltd) equipped with four detectors and multi pinhole collimation (APT62). Mice were anesthetized with isoflurane (4% for induction, and 1.5% during images acquisition) and placed in a Multicell Mouse L bed (Mediso Ltd) with temperature control (37 °C). Nucline software (Nucline 3.00.018, Mediso Ltd) was used for image acquisitions and reconstructions. CT parameters: helical scan with 480 projections (300 ms *per* projection), 50 kV, 590 μA, pitch 1.0, binning 1:4 and field of view: max. SPECT acquisitions parameters: images were acquired within the CT scan range, with resolution set to “standard”. The time *per* projection was determined in accordance with the detected radioactivity (most frequently used: 30 s). SPECT images reconstruction were conducted using TeraTomo3D (Nucline v3.00.018, Mediso Ltd) with a normal dynamic range. Regularization filters, reconstruction resolution and iterations were set to “medium”. Additional corrections were performed during reconstruction: Monte Carlo correction quality was set to “high”; Attenuation: based on CT attenuation map and scatter corrections; Activity decay correction: during acquisition time lapse.

^99m^Tc-NTP 15-5 activity was quantified in a volume of interest delineated over the operated (pathologic) and non-operated (contralateral) knees. The activity ratios between pathologic over contralateral knees were calculated for each animal at each time point.

### Histology

Histology was performed at 12 and/or 24 weeks (W) after DMM surgery. After sacrifice, whole knee joints were dissected free of soft tissues. The murine knee joints were fixed in 4% paraformaldehyde (PFA; pH 7.4) for 48 h and decalcified with ethylenediaminetetraacetic acid (EDTA) 0.5 M for 15 days at room temperature (RT) with the solution changed twice a week. After dehydration in a graded series of alcohol, murine knees were embedded in paraffin at 60 °C. Serial Sects. (5 µm thick) were cut in the medial femorotibial compartment using a Polycut E microtome Leica RM2135 (Leica, Wetzlar, Germany) and then mounted on slides. Osteoarthritis scoring was based on Safranin O-Fast green (0.02% fast green for 30 min, 1% acetic acid for 10 s, and 1.5% Safranin O for 3 min) stained-sections according to OARSI (The Osteoarthritis Research Society International) recommendations with a total severity score ranging from 0 to 6^24^. For each animal, the OA score was the highest score obtained at one of the 3 levels. All sections of each model were blindly scored by the same 2 readers. All images were taken using a Olympus DP73 microscope.

### Proteoglycan content of whole knee extract

Proteoglycan content of DMM mice knees was measured straight after ^99m^Tc-NTP 15-5 imaging, 5 weeks post-surgery. Hind legs were removed, and knees were isolated and incubated for 16 h at 65 °C with ethylenediaminetetraacetic (EDTA) buffer solution containing Papain 0.60 mg/ml. Digests were processed for proteoglycan content determination using the 1,9-dimethylmethylene blue dye-binding method (Blyscan kit, Biocolor)^47^. Proteoglycan ratio was calculated as follows: PK/CK ratio = PG content (operated knee)/PG content (contralateral knee).

### Statistics

A Student t-test was used to compare groups. A P-value < 0.05 represented uncertainty with a 95% confidence interval. Data are expressed as the mean ± standard error of the mean (SEM). Statistical analyses were performed using GraphPad Prism 8 (GraphPad Software, San Diego, CA, USA).

### Human and animal right

Animal experiments were conducted in compliance with the 2010/63/UE European Directive and with the relevant guidelines and regulations. In addition, all experiments were authorized by the French Ministry of Research after approval by the local ethical committee of Clermont-Ferrand (CEMEAA n°002, approval n°04,623.02 and n°12,160-2017111317154154v3). The study reporting in this manuscript follows the recommendations in the ARRIVE guidelines.

## References

[1] Kloppenburg M, Berenbaum F (2020). Osteoarthritis in review 2019: epidemiology and therapy. Osteoarthr. Cartil..

[2] Temple MM (2007). Age- and site-associated biomechanical weakening of human articular cartilage of the femoral condyle. Osteoarthr. Cartil..

[3] Aigner T, McKenna L (2002). Molecular pathology and pathobiology of osteoarthritic cartilage. CMLS Cell. Mol. Life Sci..

[4] Katz JN (2020). Disease modification in osteoarthritis; pathways to drug approval. Osteoarthr. Cartil..

[5] Oo WM, Hunter DJ (2019). Disease modification in osteoarthritis: are we there yet?. Clin. Exp. Rheumatol..

[6] Ghouri A, Conaghan PG (2020). Prospects for therapies in osteoarthritis. Calcif. Tissue Int..

[7] Davies PS, Graham SM, Macfarlane RJ, Leonidou A, Mantalaris A, Tsiridis E (2013). Disease-modifying osteoarthritis drugs: in vitro and in vivo data on the development of DMOADs under investigation. Expert. Opin. Investig. Drugs..

[8] Ding C, Zhang Y, Hunter D (2013). Use of imaging techniques to predict progression in osteoarthritis. Curr. Opin. Rheumatol..

[9] Moore EE (2005). Fibroblast growth factor-18 stimulates chondrogenesis and cartilage repair in a rat model of injury-induced osteoarthritis. Osteoarthr. Cartil..

[10] Reker D (2017). Sprifermin (rhFGF18) modulates extracellular matrix turnover in cartilage explants ex vivo. J. Transl. Med..

[11] Reker D (2020). Sprifermin (rhFGF18) versus vehicle induces a biphasic process of extracellular matrix remodeling in human knee OA articular cartilage ex vivo. Sci. Rep..

[12] Hochberg MC (2019). Effect of intra-articular sprifermin vs placebo on femorotibial joint cartilage thickness in patients with osteoarthritis: the FORWARD randomized clinical trial. JAMA.

[13] Eckstein F (2021). Long-term structural and symptomatic effects of intra-articular sprifermin in patients with knee osteoarthritis: 5-year results from the FORWARD study. Ann. Rheum. Dis..

[14] Guehring H (2021). The effects of sprifermin on symptoms and structure in a subgroup at risk of progression in the FORWARD knee osteoarthritis trial. Semin. Arthritis Rheum..

[15] Kiani C, Chen L, Wu YJ, Yee AJ, Yang BB (2002). Structure, and function of aggrecan. Cell. Res..

[16] Miot-Noirault E (2007). Early detection and monitoring of cartilage alteration in the experimental meniscectomized guinea pig model of osteoarthritis by ^99m^Tc-NTP 15-5 scintigraphy. Eur. J. Nucl. Med. Mol. Imaging.

[17] Miot-Noirault E, Vidal A, Auzeloux P, Madelmont JC, Maublant J, Moins N (2008). First *in vivo* SPECT imaging of mouse femorotibial cartilage using ^99m^Tc-NTP 15-5. Mol. Imaging.

[18] Cachin F (2011). First *ex vivo* study demonstrating that ^99m^Tc-NTP 15-5 radiotracer binds to human articular cartilage. Eur. J. Nucl. Med. Mol. Imaging.

[19] Miot-Noirault E (2012). In vivo experimental imaging of osteochondral defects and their healing using ^99m^Tc-NTP 15-5 radiotracer. Eur. J. Nucl. Med. Mol. Imaging.

[20] Miot-Noirault E, Vidal A, Auzeloux P, Peyrode C, Madelmont JC, Chezal JM (2012). *In vivo* scintigraphic imaging of proteoglycans. Methods Mol. Biol..

[21] Khairnar A (2015). ^99m^Tc-NTP 15-5 imaging for cartilage involvement in experimental rheumatoid arthritis comparison with routinely used molecular imaging methods and sensitivity to chronic NSAID treatment. J. Nucl. Med..

[22] Lim NH, Wen C, Vincent TL (2020). Molecular and structural imaging in surgically induced murine osteoarthritis. Osteoarthritis Cartilage.

[23] Fang H (2018). Early changes of articular cartilage and subchondral bone in the DMM mouse model of osteoarthritis. Sci. Rep..

[24] Glasson SS, Chambers MG, Van Den Berg WB, Little CB (2010). The OARSI histopathology initiative - recommendations for histological assessments of osteoarthritis in the mouse. Osteoarthr. Cartil..

[25] Ollier M (2001). Joint scintigraphy in rabbits with 99mTc-N-[3-(triethylammonio)propyl]-15ane-N5, a new radiodiagnostic agent for articular cartilage imaging. J. Nucl. Med..

[26] Adams MPE, Matyas JR, Huang D, Dourado GS (1995). Expression of proteoglycans and collagen in the hypertrophic phase of experimental osteoarthritis. J. Rheumatol. Suppl..

[27] Venn G, Billigham ME, Hardingham TE (1995). Increased proteoglycan synthesis in cartilage in experimental canine osteoarthritis does not reflect a permanent change in chondrocyte phenotype. Arthr. Rheum..

[28] Wei L, Svensson O, Hjerpe A (1998). Proteoglycan turnover during development of spontaneous osteoarthrosis in guinea pigs. Osteoarthr. Cartil..

[29] Lorenzo P, Bayliss MT, Heinegard D (2004). Altered patterns and synthesis of extracellular matrix macromolecules in early osteoarthritis. Matrix Biol..

[30] Peng Z (2021). The regulation of cartilage extracellular matrix homeostasis in joint cartilage degeneration and regeneration. Biomater..

[31] Maly K, Sastre EA, Farrel E, Meurer A, Zaucke F (2021). COMP and TSP-4: functional roles in articular cartilage and relevance in osteoarthritis. Int. J. Mol. Sci..

[32] Lohmander LS (2014). Intraarticular Sprifermin (recombinant human fibroblast growth factor 18) in knee osteoarthritis: A randomized, double-blind, placebo-controlled trial. Arthr. Rheumatol..

[33] Eckstein F, Wirth W, Guermazi A, Maschek S, Aydemir A (2015). Intraarticular sprifermin not only increases cartilage thickness, but also reduces cartilage loss: location-independent post-hoc analysis using magnetic resonance imaging. Arthr. Rheumatol..

[34] Eckstein F, Kraines JL, Aydemir A, Wirth W, Maschek S, Hochberg MC (2020). Intra-articular sprifermin reduces cartilage loss in addition to increasing cartilage gain independant of location in the femorotibial joint: post-hoc analysis of a randomised, placebo-controlled phase II clinical trial. Ann. Rheu. Dis..

[35] Conaghan PG, Hunter DJ, Maillefert JF, Reichmann WM, Losina E (2011). Summary and recommendations of the OARSI FDA Osteoarthritis assessment of structural change working group. Osteoarthr. Cartil..

[36] Roemer FW, Kwoh CK, Hayashi D, Felson DT, Guermazi A (2018). Perspectives: the role of radiography and MRI in determining patient eligibility for clinical trials of knee osteoarthritis. Nat. Rev. Rheum..

[37] Bijlsma JW, Berenbaum F, Lafeber FP (2011). Osteoarthritis: an update with relevance for clinical practice. Lancet.

[38] Hayashi D, Roemer FW, Guermazi A (2019). Imaging of osteoarthritis by conventional radiography, MR imaging, PET computed tomography, and PET-MR imaging. PET Clin..

[39] Watkins L (2021). Assessment of quantitative [^18^F]Sodium fluoride PET measures of knee subchondral bone perfusion and mineralization in osteoarthritic and healthy subjects. Osteoarthr. Cartil..

[40] Nakamura H (2007). Positron emission tomography with 18F-FDG in osteoarthritic knee. Osteoarthr. Cartil..

[41] Kim J (2017). Maximum standardized uptake value of quantitative bone SPECT-CT in patients with medial compartment osteoarthritis of the knee. Clin. Radiol..

[42] Piscaer TM (2011). Imaging of activated macrophages in experimental osteoarthritis using folate-targeted animal single photon emission computed tomography/computed tomography. Arthr. Rheumat..

[43] De Visser HM (2018). Imaging of folate receptor expressing macrophages in the rat Groove model of osteoarthritis: using a new DOTA-Folate conjugate. Cartilage.

[44] Sobal G, Velusamy K, Kosik S, Menzel J, Hacker M, Pagitz M (2016). Preclinical evaluation of ^99m^Tc labeled chondroitin sulfate for monitoring of cartilage degeneration in osteoarthritis. Nucl. Med. Biol..

[45] Laasanen MS (2003). Biochemical properties of knee articular cartilage. Biotechnology.

[46] Ma HL, Blanchet TJ, Peluso D, Hopkins B, Morris EA, Glasson SS (2007). Osteoarthritis severity is sex dependent in a surgical mouse model. Osteoarthr. Cartil..

[47] Farndale RW, Buttle DJ, Barrett AJ (1986). Improved quantitation, and discrimination of sulphated glycosaminoglycans by use of dimethylmethylene blue. Biochim. Biophys. Acta.

